# Deficiency of ECHS1 causes mitochondrial encephalopathy with cardiac involvement

**DOI:** 10.1002/acn3.189

**Published:** 2015-03-13

**Authors:** Tobias B Haack, Christopher B Jackson, Kei Murayama, Laura S Kremer, André Schaller, Urania Kotzaeridou, Maaike C de Vries, Gudrun Schottmann, Saikat Santra, Boriana Büchner, Thomas Wieland, Elisabeth Graf, Peter Freisinger, Sandra Eggimann, Akira Ohtake, Yasushi Okazaki, Masakazu Kohda, Yoshihito Kishita, Yoshimi Tokuzawa, Sascha Sauer, Yasin Memari, Anja Kolb-Kokocinski, Richard Durbin, Oswald Hasselmann, Kirsten Cremer, Beate Albrecht, Dagmar Wieczorek, Hartmut Engels, Dagmar Hahn, Alexander M Zink, Charlotte L Alston, Robert W Taylor, Richard J Rodenburg, Regina Trollmann, Wolfgang Sperl, Tim M Strom, Georg F Hoffmann, Johannes A Mayr, Thomas Meitinger, Ramona Bolognini, Markus Schuelke, Jean-Marc Nuoffer, Stefan Kölker, Holger Prokisch, Thomas Klopstock

**Affiliations:** 1 Institute of Human Genetics, Technische Universität München81675, Munich, Germany; 2 Institute of Human Genetics, Helmholtz Zentrum München, German Research Center for Environmental Health85764, Neuherberg, Germany; 3 Institute of Clinical Chemistry and University Children's Hospital, University of Bern3010, Bern, Switzerland; 4 Department of Metabolism, Chiba Children's HospitalChiba, 266-0007, Japan; 5 Division of Human Genetics, Department of Pediatrics, University of Bern3010, Bern, Switzerland; 6 Divisions of Inherited Metabolic Disease and Neuropediatrics, Department of General Pediatrics, University Hospital HeidelbergD-69120, Heidelberg, Germany; 7 Department of Pediatrics, Nijmegen Center for Mitochondrial Disorders, Radboud University Center6525 GA, Nijmegen, The Netherlands; 8 Department of Neuropediatrics and NeuroCure Clinical Research Center, Charité Universitätsmedizin Berlin13353, Berlin, Germany; 9 Department of Pediatrics, Birmingham Children's HospitalBirmingham, B4 6NH, United Kingdom; 10 Department of Neurology, Friedrich-Baur-Institute, Ludwig-Maximilians-University80336, Munich, Germany; 11 Department of Pediatrics, Klinikum Reutlingen72764, Reutlingen, Germany; 12 Department of Pediatrics, Faculty of Medicine, Saitama Medical UniversitySaitama, 350-0495, Japan; 13 Division of Translational Research, Research Center for Genomic Medicine, Saitama Medical UniversitySaitama, 350-1241, Japan; 14 Division of Functional Genomics & Systems Medicine, Research Center for Genomic Medicine, Saitama Medical UniversitySaitama, 350-1241, Japan; 15 Max-Planck-Institute for Molecular Genetics, Otto-Warburg Laboratory14195, Berlin, Germany; 16 Wellcome Trust Sanger InstituteHinxton, Cambridge, CB10 1SA, United Kingdom; 17 Department of Neuropediatrics, Children's Hospital of Eastern Switzerland St.Gallen9006, St. Gallen, Switzerland; 18 Institute of Human Genetics, University of Bonn53127, Bonn, Germany; 19 Institut für Humangenetik, Universitätsklinikum Essen, Universität Duisburg-Essen45122, Essen, Germany; 20 Wellcome Trust Centre for Mitochondrial Research, Institute of Neuroscience, The Medical School, Newcastle UniversityNewcastle upon Tyne, NE2 4HH, United Kingdom; 21 Department of Pediatrics, Friedrich-Alexander-University of Erlangen-Nürnberg91054, Erlangen, Germany; 22 Department of Pediatrics, Paracelsus Medical University Salzburg5020, Salzburg, Austria; 23 Munich Cluster for Systems Neurology (SyNergy)80336, Munich, Germany; 24 DZNE – German Center for Neurodegenerative Diseases80336, Munich, Germany

## Abstract

**Objective:**

Short-chain enoyl-CoA hydratase (ECHS1) is a multifunctional mitochondrial matrix enzyme that is involved in the oxidation of fatty acids and essential amino acids such as valine. Here, we describe the broad phenotypic spectrum and pathobiochemistry of individuals with autosomal-recessive ECHS1 deficiency.

**Methods:**

Using exome sequencing, we identified ten unrelated individuals carrying compound heterozygous or homozygous mutations in *ECHS1*. Functional investigations in patient-derived fibroblast cell lines included immunoblotting, enzyme activity measurement, and a palmitate loading assay.

**Results:**

Patients showed a heterogeneous phenotype with disease onset in the first year of life and course ranging from neonatal death to survival into adulthood. The most prominent clinical features were encephalopathy (10/10), deafness (9/9), epilepsy (6/9), optic atrophy (6/10), and cardiomyopathy (4/10). Serum lactate was elevated and brain magnetic resonance imaging showed white matter changes or a Leigh-like pattern resembling disorders of mitochondrial energy metabolism. Analysis of patients’ fibroblast cell lines (6/10) provided further evidence for the pathogenicity of the respective mutations by showing reduced ECHS1 protein levels and reduced 2-enoyl-CoA hydratase activity. While serum acylcarnitine profiles were largely normal, in vitro palmitate loading of patient fibroblasts revealed increased butyrylcarnitine, unmasking the functional defect in mitochondrial *β*-oxidation of short-chain fatty acids. Urinary excretion of 2-methyl-2,3-dihydroxybutyrate – a potential derivative of acryloyl-CoA in the valine catabolic pathway – was significantly increased, indicating impaired valine oxidation.

**Interpretation:**

In conclusion, we define the phenotypic spectrum of a new syndrome caused by ECHS1 deficiency. We speculate that both the *β*-oxidation defect and the block in l-valine metabolism, with accumulation of toxic methacrylyl-CoA and acryloyl-CoA, contribute to the disorder that may be amenable to metabolic treatment approaches.

## Introduction

**Figure 1:**
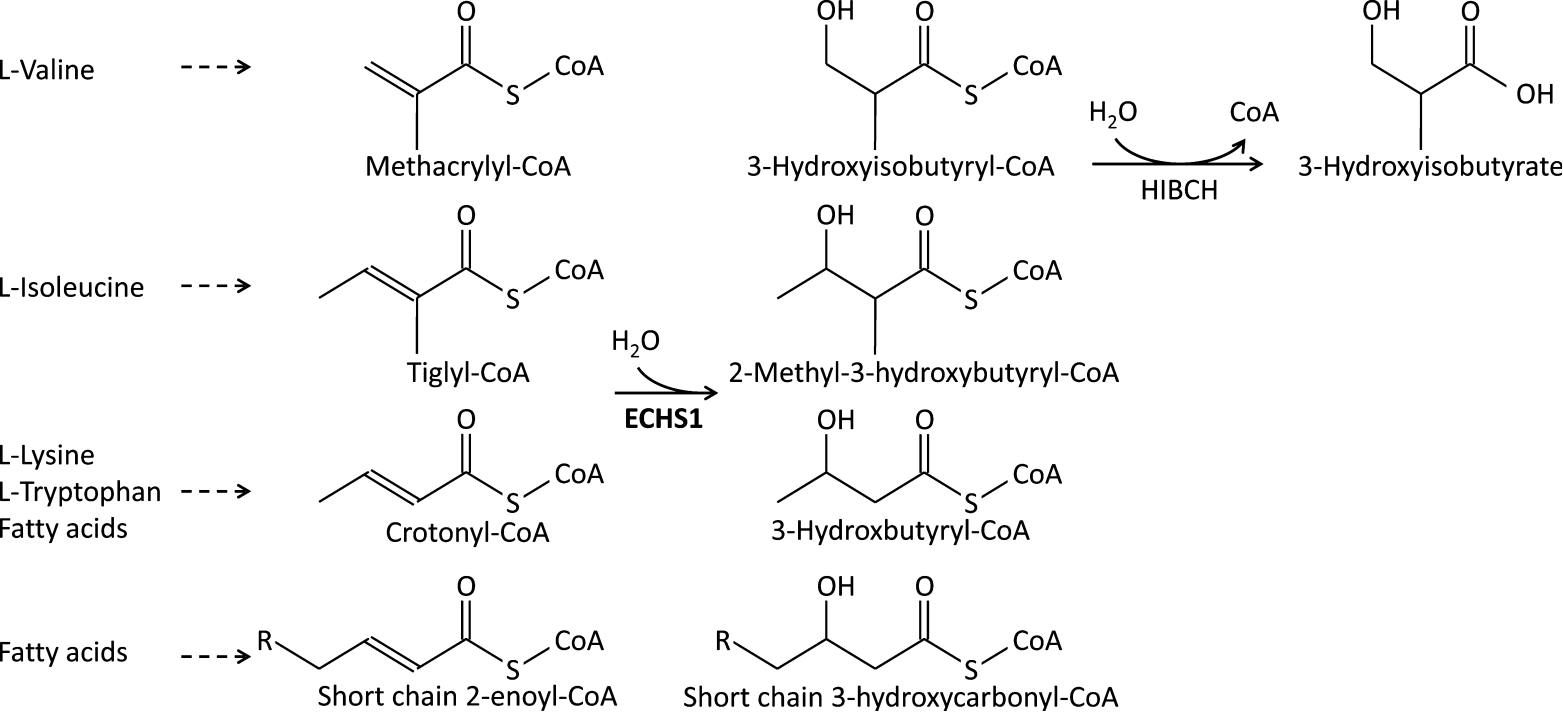
Short-chain enoyl-CoA hydratase (ECHS1) functions. Proposed functions of ECHS1 in the mitochondrial amino acid and fatty acid metabolism with illustration of the level of HIBCH (3-hydroxyisobutyryl-CoA hydrolase) deficiency.

Short-chain enoyl-CoA hydratase (ECHS1, synonym: crotonase, EC 4.2.1.17), encoded by *ECHS1* (cytogenetic location: 10q26.3; GenBank accession number: NM_004092.3; OMIM*602292), is a mitochondrial matrix enzyme that catalyzes the second step of the *β*-oxidation spiral of fatty acids, that is, the hydration of chain-shortened *α*,*β*-unsaturated enoyl-CoA thioesters to produce *β*-hydroxyacyl-CoA.^1^ For each turn of this spiral pathway, one acetyl-CoA molecule is released and utilized for either the formation of citrate (tricarboxylic acid [TCA] cycle) or ketone bodies (ketogenesis). Decreased activity of mitochondrial *β*-oxidation of fatty acids thus decreases the formation of important energy substrates. Decreased formation of acetyl-CoA results in increased susceptibility to energy deficiency during catabolic states and to the dysfunction of organs that particularly rely on fatty acids and ketone bodies as their energy source (e.g., cardiac tissue). In addition, decreased formation of acetyl-CoA, and hence limited availability of acetate, hampers myelination because acetate is required for cholesterol biosynthesis. Moreover, decreased formation of acetyl-CoA may hamper posttranslational acetylation of mitochondrial proteins, a mechanism that is emerging as a critical regulator of mitochondrial function.^2^ Evidence is increasing that ECHS1 has a wide substrate specificity and thus also plays an important role in amino acid catabolism, in particular of valine, where it converts methacrylyl-CoA to (S)-3-hydroxyisobutyryl-CoA and acryloyl-CoA to 3-hydroxypropionyl-CoA (Fig.1), the fourth step of valine oxidation.^3^ Accumulation of toxic methacrylyl-CoA and acryloyl-CoA, two highly reactive intermediates that spontaneously react with sulfhydryl groups of, for example, cysteine and cysteamine, is suspected to cause brain pathology and biochemical phenotype in *β*-hydroxyisobutyryl-CoA hydrolase (HIBCH) deficiency, a disorder of the fifth step of valine oxidation with a Leigh-like phenotype and deficiency of multiple mitochondrial enzymes.^4^,^5^ Very recently, *ECHS1* mutations were reported in two siblings with Leigh disease and remarkable clinical and biochemical similarities to HIBCH deficiency.^6^ Both presented soon after birth with generalized hypotonia, poor feeding, respiratory insufficiency, and developmental delay. They suffered a severe clinical course and died at the age of 4 and 8 months.

Here, we report 10 unrelated individuals, identified by exome sequencing, who carry compound heterozygous or homozygous *ECHS1* mutations and present with a combination of (Leigh-like) mitochondrial encephalopathy, deafness, epilepsy, optic nerve atrophy, and cardiomyopathy. This work confirms *ECHS1* mutations as a cause of mitochondrial disease, and defines the broad phenotypic spectrum of this new disorder which ranges from fatal neonatal courses to survival into adulthood.

## Patients and Methods

**Figure 2:**
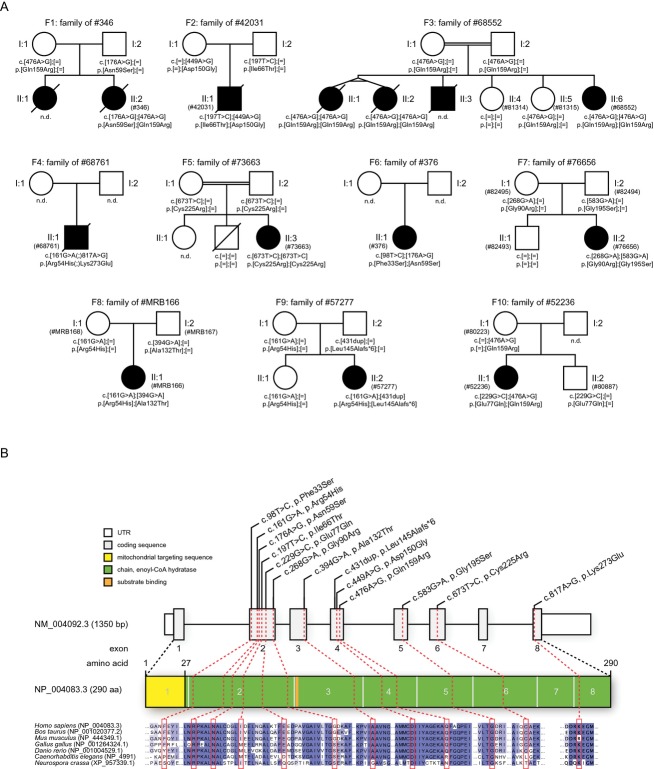
Pedigrees of investigated families and short-chain enoyl-CoA hydratase (*ECHS1*) structure and conservation of identified mutations. (A) Pedigrees of 10 families with mutations in *ECHS1*. Mutation status of affected (closed symbols) and unaffected (open symbols) family members. (B) Gene structure of *ECHS1* with known protein domains of the gene product and localization and conservation of amino acid residues affected by mutations. Intronic regions are not drawn to scale.

**Figure 3:**
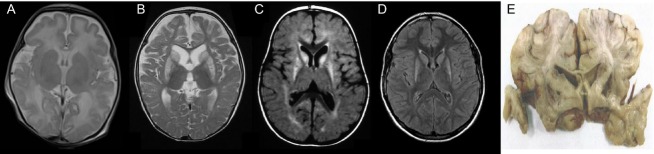
Spectrum of brain MRI and autopsy changes in ECHS1 patients. (A) MRI (T2) at age 8 days in individual F1, II:2 showing widespread diffuse white matter changes and brain atrophy. (B) MRI (T2) at age 8 months in individual F6, II:1 (#376) showing brain atrophy and bilateral symmetric signal hyperintensity in *caput nucleus caudatus* and *putamen*. (C) MRI (FLAIR) at age 2.2 years in individual F9, II:2 (#57277) showing increased signal in *putamen, globus pallidus* and *caput nucleus caudatus*. (D) MRI (FLAIR) at age 15 years in individual F10, II:1 (#52236) showing bilateral symmetric signal hyperintensity in *caput nucleus caudatus* and *putamen*. (E) Autopsy at age 11 months in individual F2, II:1 (#42031) showing necrotizing encephalopathy of the caudate and lenticular nuclei. MRI, magnetic resonance imaging; ECHS1, short-chain enoyl-CoA hydratase.

Written informed consent was obtained from all patients investigated or their guardians and the ethics committee of the Technische Universität München approved the study. The patients tested positive for *ECHS1* mutations are part of a large cohort of cases with suspected mitochondrial disorders. DNA samples have been collected for genetic analyses in three different centers including Bern (Switzerland, 47 cases including family F2), Saitama (Japan, 180 cases including families F1 and F6), and Munich (Germany, 435 cases including families F3-5 and 7-10; Fig.2). Clinical and biochemical findings of *ECHS1* mutation-positive patients are summarized in Table1 and representative abnormal magnetic resonance imaging (MRI) findings are shown in Figure3. In addition, we report on four older siblings who have died undiagnosed with a clinical picture similar to the mitochondrial encephalocardiomyopathy described in their younger siblings with a confirmed diagnosis of ECHS1 deficiency.

**Table 1:** Genetic and clinical findings in patients with *ECHS1* mutations

ID	Sex	*ECHS1* mutations	Biochemical investigations		Clinical and biochemical features											
		cDNA (NM_004092.3) protein (NP_004083.3)	Analysis	Result	AO	Course	Neuroimaging (MRI, MRS)	Hearing loss	Optic atrophy	Devel. delay	Epilepsy	Dystonia	Cardio-myopathy	Elevated lactate	2-methyl-2,3-dihydroxybutyrate	Others
F1, II:2 #346	F	c.[176A>G];[476A>G] p.[Asn59Ser];[Gln159Arg]	RCCI-V^2^	CI mildly↓	Birth	Died at age 4 m	At age 8 d: white matter changes, brain atrophy (Fig.3A), more prominent until age 58 d	Yes	n.k.	n.k.	Yes	n.k.	HCM	Yes	n.d.	Unrelated parents, 1 older sister died at age 1 m
F2, II:1 #42031	M	c.[197T>C];[449A>G] p.[Ile66Thr];[Asp150Gly]	RCCI-V^+^^1^ PDHc^+^^1^ Substrate oxidation^1^	Normal Normal Pyruvate↓	Birth	Died at age 11 m	At age 17 d: normal myelinisation, symmetrical punctiform hyperintensities in centrum semiovale MRS: lactate ↑	Yes	Yes	Yes	Yes	Yes	HCM	Yes	229-fold	Autopsy revealed subacute necrotizing encephalopathy (Fig.3E) and massive left ventricular hypertrophy.
F3, II:6 #68552	F	c.[476A>G];[476A>G] p.[Gln159Arg];[Gln159Arg]	n.d.	n.d.	Birth	Died at age 2.3 y	Symmetrical white matter changes	n.k.	n.k.	Yes	Yes	Yes	n.k.	Yes	n.d.	Consanguineous parents, 3 older siblings died before age 2 y, RCCI defect in muscle in tow of them
F4, II:1 #68761	M	c.[161G>A(;)817A>G] p.[Arg54His(;)Lys273Glu]	RCCI-V ATP production	Normal Decreased	Birth	Died at age 7.5 y	At age 4 y: extensive brain atrophy MRS: normal lactate	n.k.	n.k.	Yes	Yes	Yes	No	n.k.	n.d.	Died in the course of a pulmonary infection
F5, II:3 #73663	F	c.[673T>C];[673T>C] p.[Cys225Arg];[Cys225Arg]	RCCI-V PDHc Substrate oxidation	Normal Normal Normal	Birth	Alive at age 2 y	Delayed myelination, T_2_-hyperintense periphere white matter lesions, liquorisointense lesions in *putamen* and *pallidum*	n.k.	n.k.	Yes	Yes	No	HCM	Yes	39-fold	Consanguineous parents, a brother of this girl died at age 4 m
F6, II:1 #376	F	c.[98T>C];[176A>G] p.[Phe33Ser];[Asn59Ser]	RCCI-IV	CIV mildly↓	Birth	Alive at ag e 3 y	Symmetrical bilateral signal abnormalities in basal ganglia(Fig.3B)	Yes	n.k.	Yes	Yes	n.k.	DCM	Yes	n.d.	n.a.
F7, II:2 #76656	F	c.[268G>A];[583G>A] p.[Gly90Arg];[Gly195Ser]	RCCI-IV^1^ PDHc^1^	Normal Normal	2 y	Alive at age 5 y	At age 2 y: no atrophy, but signal hyperintensities of *putamen, globus pallidus, nucleus caudatus* and periventicular white matter MRS: lactate ↑	Yes	No	Yes	No	Yes	n.k.	n.d.	sixfold	n.a.
F8, II:1 #MRB166	F	c.[161G>A];[394G>A] p.[Arg54His];[Ala132Thr]	n.d.	n.d.	1 y	Alive at age 8 y	n.a.	Yes	No	Yes	No	No	n.k.	Yes	n.d.	Gastroschisis, truncal ataxia, muscular hypotonia, increased muscle tonus, cochlear implant
F9, II:2 #57277	F	c.[161G>A];[431dup] p.[Arg54His];[Leu145Alafs^*^6]	RCCI-IV Pyruvate oxidation	Normal Normal	Birth	Alive at age 16 y	At age 1.5 y: increased T2-signal intensity in *putamen* and *globus pallidus* which became more prominent until age 2.2 years (Fig.3C) MRS: lactate ↑	Yes	Yes	Yes	No	Yes	No	Yes	n.d.	Communicates through a voice computer at age 16 y
F10, II:1 #52236	F	c.[229G>C];[476A>G] p.[Glu77Gln];[Gln159Arg]	RCCI-IV	Normal	11 m	Alive at age 31 y.	At age 15 y: no atrophy, but signal hyperintensities in *nucleus caudatus* and *putamen* (Fig.3D) MRS: normal lactate	Yes	Yes	Yes	Yes	Yes	No	Yes	Normal	Spastic tetraparesis, confined to wheelchair from age 9.5 y

ECHS1, short-chain enoyl-CoA hydratase; MRI, magnetic resonance imaging; AO, age of onset; m, months; y, years; n.a. not applicable; HCM, hypertrophic cardiomyopathy; DCM, dilated cardiomyopathy; n.d. not determined; n.k., not known, Mitochondrial respiratory chain complexes (RCC) in muscle: I, NADH:CoQ oxidoreductase; II, succinate dehydrogenase; II + III, succinate:cytochrome c reductase; IV, cytochrome c oxidase (COX).

Enzyme activities were determined in muscle biopsies if not a stated otherwise (^1^Investigated in fibroblast cell lines; ^2^Investigated in liver) and normalized to citrate synthase (CS).

Regarding terminology, we avoided the term “Leigh syndrome” or “Leigh-like syndrome” for the whole group of our patients with ECHS1 deficiency, because these are ill-defined entities and many of our patients did not fulfill the criteria suggested by Rahman et al.^7^ In those individual cases that fulfilled the definition by Rahman, we preferred to use the more neutral term “Leigh-like syndrome” or “Leigh-like pattern in MRI”.

### Case reports

Patient #346 (F1, II:2, c.[176A>G];[476A>G], p.[Asn59Ser];[Gln159Arg]), a girl, was born after a normal pregnancy at 39 weeks of gestation with normal birth measurements (weight 2935 g, length 50.5 cm) as the second child of unrelated Japanese and American parents. Soon after birth, she was admitted to a neonatal medical center for severe respiratory and cardiac failure with hypertrophic cardiomyopathy (HCM) and suspected deafness. There was profound lactic acidosis in blood (21–43 mmol/L, lower limit of normal 1.8 mmol/L), but metabolic profiling (amino acid analysis, urine organic acid analysis, acylcarnitine analysis) was unremarkable. Brain MRI at day 8 showed low intensity in cerebral white matter, and moderate brain atrophy at day 58. She died at the age of 4 months and autopsy was performed. Respiratory chain analysis showed mild deficiency of complex I in liver, but normal activities in muscle and heart. Her older sister died due to respiratory failure and severe lactic acidosis on her first day of life.

Patient #42031 (F2, II:1, c.[197T>C];[449A>G], p.[Ile66Thr];[Asp150Gly]), a boy, is the first child of healthy nonconsanguineous Swiss parents. After a normal pregnancy, he was born at 42 weeks of gestation with normal birth measurements. Postnatally, he developed lactic acidosis and neonatal seizures. Analysis of fibroblasts showed reduced pyruvate oxidation compatible with a complex I or pyruvate dehydrogenase defect, whereas enzymatic activities of the respiratory chain in muscle and fibroblasts were normal. These findings led to a therapeutic trial with ketogenic diet. The diet was stopped after a few months as no clinical response was observed. At 5 months of age, he had severely delayed motor development, hardly any head control, severe truncal hypotonia and intermittent episodes of opisthotonus. There was no reaction to visual or auditory stimuli. Despite gastric tube feeding, the child was severely underweight (−2.8 SD), of short stature (−3.4 SD), and microcephalic (−4.9 SD).

Laboratory investigation revealed persistently elevated lactate (2.4–6.0 mmol/L), mildly elevated CK and repeatedly normal acylcarnitines. Repeated electroencephalograms did not show epileptic discharges. Brain MRI at age 17 days showed normal myelinisation but symmetrical punctiform hyperintensities in the centrum semiovale. MR spectroscopy of basal ganglia showed elevated lactate. Ophthalmological examination suggested bilateral optic atrophy, and acoustic evoked potentials confirmed severe sensorineural deafness. At the age of 11 months, the child was found dead in his bed. Autopsy revealed morphological and histological findings of subacute necrotizing encephalopathy (Fig.3E) and massive left ventricular hypertrophy.

Patient #68552 (F3, II:6, c.[476A>G];[476A>G], p.[Gln159Arg];[Gln159Arg]), a girl, is the sixth child of first cousins of Pakistani origin. Shortly after birth, the infant was found hypotonic with poor feeding and with high lactate (5.1 mmol/L). She was extremely irritable and had episodes of stiffness but electroencephalography (EEG) was normal. Even so, the baby was started on antiepileptic drug therapy as well as on baclofen. She was fed continuously by nasogastric tube. She showed no developmental progress nor developed any meaningful interaction with her environment and her irritability worsened episodically. Palliative care was instituted and she died aged 2 years and 4 months.

Brain MRI showed symmetrical white matter changes with a periventricular focus and extension into the subcortical areas of the frontal and parietal lobes. The thalami as well as the caudate and lentiform nuclei appeared normal. MR spectroscopy of basal ganglia showed no obvious lactate peak. Neonatal adrenoleukodystrophy, biotinidase deficiency, Krabbe disease, GM1 gangliosidosis and metachromatic leukodystrophy were excluded biochemically. There were no significant abnormalities of acylcarnitines, organic acids, glycosaminoglycans, oligosaccharides or amino acids (except a raised alanine in keeping with lactic acidosis). Invasive investigations such as muscle biopsy were refused.

Molecular genetic screening demonstrated a maternally transmitted m.1555A>G *MTRNR1* mtDNA mutation that is characteristically associated with aminoglycoside-induced hearing loss. This was not felt to be responsible for the patient's condition.

Three older siblings of this patient had congenital lactic acidosis and died between the ages of 1 and 2 years. The first two affected children were identical twin girls and the third affected child was a boy. The history is identical in all three children. They were all born at term via normal vaginal delivery. There was no history of birth asphyxia and they were apparently well soon after birth. They fed well initially but became symptomatic aged between 1 and 2 days with generalized tonic-clonic seizures. Subsequently, they had poor feeding and severe developmental delay. The seizures were recurrent but well controlled with anticonvulsants. They were tube-fed from the first few days of life as they were unable to suck and swallow effectively. They had severe developmental delay from the outset and showed little evidence of development during infancy. They always had a poor head control, poor eye contact, and a poor smile. They were never able to reach out and their hearing was possibly impaired. There was no history of dystonia and they were said to be very hypertonic. There were no breathing problems reported. They apparently had no renal tubular acidosis but they required some treatment with bicarbonate for metabolic acidosis (lactate levels of ∼5.0 mmol/L). There was no history of cardiac or liver involvement. The twin girls died aged 2 years and the boy died aged 1 year. In the twin girls, a diagnosis of complex I deficiency was established on muscle biopsy respiratory chain enzyme analysis. Testing of *ECHS1* performed on newborn screening bloodspots of the twin older siblings (F3, II:1 and II:2) confirmed that they were also homozygous for the c.476A>G, p.Gln159Arg variant. The children were treated with sodium bicarbonate, anticonvulsants, and a mitochondrial vitamin cocktail as well as nasogastric tube feeding. The family has two other healthy girls, now teenagers.

Patient #68761 (F4, II:1, c.[161G>A(;)817A>G] p.[Arg54His(;)Lys273Glu]), a boy, is the first child to healthy unrelated parents from The Netherlands. After a normal pregnancy, he was born at gestational age 39 + 1 weeks by Cesarean section on maternal indication with a birth weight of 3990 g. Apgar scores were 8 after 1 min and 9 after 5 min. Mild generalized muscular hypotonia was observed upon birth which was accompanied by feeding problems until the age of 5 months. Thereafter, his clinical condition declined and he developed severe encephalopathy with hardly any spontaneous movements of the head and trunk, swallowing problems, and episodes of inconsolable crying. He depended on tube feeding and suffered from epilepsy with hypsarrhythmia and multifocal epileptic activity in the EEG at the age of 1 and 3 years.

On physical examination, he was unable to make contact or follow objects, showed virtually no spontaneous movements, but only dystonic movements of arms and legs. Over the course of the disease axial hypotonia and hypertonia of the limbs persisted, dystonic movements diminished, and he did not make any developmental progress. Laboratory tests showed chronic iron deficiency leading to anemia. Physical examination at the age of 6 years showed microcephaly, scoliosis, and muscular hypotonia. The child died at the age of 7.5 years due to respiratory insufficiency in the course of a pulmonary infection.

Brain MRI at the age of 1 year showed atrophy of caudate nuclei, corpus callosum, mesencephalon, and pons. These changes were progressive at age 4 years showing extensive white and gray matter brain atrophy, mainly in frontal and temporal lobes bilaterally with subsequent widening of the subarachnoid space and of ventricular system. Brain MR spectroscopy showed an overall decrease in NAA being most pronounced in the basal ganglia but no elevation of lactate.

Cardiac ultrasound at the ages of 1 and 4 years showed no structural or functional abnormalities. Biochemical analysis of a skeletal muscle specimen showed normal citrate synthase (CS)-adjusted activities of respiratory chain complexes I–V but a decreased overall ATP production. Pathogenic mutations of the mitochondrial DNA were excluded by Sanger sequencing of DNA from muscle.

Patient #73663 (F5, II:3, c.[673T>C];[673T>C], p.[Cys225Arg];[Cys225Arg]), a girl, was born at term as the third child of consanguineous parents after normal pregnancy and spontaneous vaginal delivery. On day 5, she was admitted to hospital due to rapid loss of body weight (24% below birth weight) and severe metabolic acidosis (pH 6.86). Lactate, alanine, and ketone bodies were also strongly increased leading to the suspicion of an inherited disorder of mitochondrial energy metabolism. Following this neonatal decompensation she showed a severe global development delay, severe generalized spastic tetraparesis, myoclonic epilepsy, and HCM. At age 16 months, she had a cardiac arrest following a diagnostic muscle and skin biopsy. She survived after 45 min of cardiopulmonary resuscitation but several complications followed this event (ARDS, sepsis, aspiration pneumonia, acute renal failure, and acute hepatic failure). At age 2.3 years she does not show active movement of arms and legs and is not able to sit, stand or walk. She neither speaks nor fixes or follows persons and objects. She reacts to voices and noise.

Cranial MRI performed at age 13 months showed delayed myelination, a thin corpus callosum, and T2 signal abnormalities in the periventricular white matter. Lesions in *putamen* and *globus pallidus* were also found. MR spectroscopy of gray and white matter was normal. Metabolic work-up revealed elevated serum lactate (up to 8.5 mmol/L), moderately elevated plasma alanine (up to 630 *μ*mol/L), slightly elevated ethylmalonic acid in urine (60 mmol/mol creatinine), and intermittently low plasma ketone bodies in preprandial state. Hyperuricemia was found. Acylcarnitine profiling was normal. Radiometric and single enzyme analysis of OXPHOS in frozen muscle tissue did not confirm the suspected diagnosis of a mitochondrial disorder.

A brother of this girl has died at age 4 months during an acute decompensation similar to that described above. He also had severe developmental delay, elevated lactate and metabolic acidosis. Similar to his younger sister, liver, skin, and muscle biopsy did not confirm a respiratory chain defect in this child.

Patient #376 (F6, II:1, c.[98T>C];[176A>G], p.[Phe33Ser];[Asn59Ser]), a girl, was born after an uneventful pregnancy at 39 weeks of gestation with normal birth measurements (weight 3124 g, length 51 cm) as the first child of unrelated Japanese parents. At the age of 2 days, she had epileptic seizures and was treated with phenobarbitone. In her first months, developmental delay and muscular hypotonia were noted. At the age of 8 months, she developed respiratory failure and unconsciousness, and was admitted to hospital. Transient lactic acidosis in blood (8.0 mmol/L, lower limit of normal 1.8 mmol/L) was found. Metabolic profiling (amino acid analysis, urine organic acid analysis, and acylcarnitine analysis) was unremarkable. Brain CT and MRI at 8 months showed symmetrical, bilateral signal abnormalities in basal ganglia, and she was diagnosed with Leigh-like syndrome. At the age of 1 year, she developed deafness, and at the age of 3 years dilated cardiomyopathy. Muscle biopsy showed mild deficiency of complex IV.

Patient #76656 (F7, II:2, c.[268G>A];[583G>A], p.[Gly90Arg];[Gly195Ser]), a girl, is the child of healthy unrelated German parents. After a normal pregnancy, she was born at term with normal birth measurements. The early motor development was normal, and she started walking at the age of 14 months. The girl was referred at age 2 years because of stiffness of gait and a tendency to fall. At admission, she showed muscular hypotonia, coordination problems, choreoathetotic movements, delayed speech development, and sensorineural deafness, which had been treated with hearing aids. Blood chemistry was normal including lactate, amino acids and organic acids.

Brain MRI at age 2 years showed no cerebral, cerebellar, or callosal atrophy but mild bilateral signal hyperintensities of putamen, globus pallidus, nucleus caudatus, and periventricular white matter. MR spectroscopy of basal ganglia showed mild elevation of lactate.

A muscle biopsy showed only unspecific morphologic findings, and biochemical analysis of respiratory chain activities was unremarkable.

Patient #MRB166 (F8, II:1, c.[161G>A];[394G>A], p.[Arg54His];[Ala132Thr]), a girl, is the first child of healthy unrelated German parents. Pregnancy was complicated by oligohydramnion and a diagnosis of gastroschisis. She was born by Cesarean section at 34 weeks of gestation with normal birth measurements (weight 1490 g [3rd percentile], length 41 cm [25th percentile], and head circumference 31 cm [25th percentile]). Surgical correction of gastroschisis was performed. The girl needed phototherapy for icterus neonatorum, tube feeding, and assisted ventilation. Deafness was diagnosed with 2 years of age and treated by cochlear implants. One seizure was observed at the age of 7 years. She showed truncal hypotonia, gait ataxia, and severe developmental delay (crawling at 1.5 years, sitting at 3 years, assisted walking at 5 years of age and no speech at 8 years). She has an oval face, a broad nasal tip, a large mouth, a high arched palate, and a receding chin. MLPA PWS/AS, *UBE3A*, and *MECP2* mutation analysis and microarray gave normal results.

Patient #57277 (F9, II:2, c.[161G>A];[431dup], p.[Arg54His];[Leu145Alafs*6]), a girl, is the second child of healthy nonconsanguineous parents from Germany. She was born after a normal pregnancy at 41 weeks of gestation with normal body measurements. Early motor development was delayed, and she never acquired the ability to sit or walk independently. Acquisition of language was impeded by sensorineural deafness for which she was fitted with hearing aids. At 18 months parents noted a pendular nystagmus with a rotatoric component as well as loss of central vision due to optic atrophy. Residual vision at 10 years was 0.3. From 2 years of age her motor abilities deteriorated during episodes of febrile illness and lactic acidemia when she developed a movement disorder that undulated between dystonia (opisthotonus) and chorea. Treatment with Levodopa/Carbidopa worsened her symptoms. She is presently 16 years old. Her body length is at and her weight 1 kg below the third percentile. Due to dysphagia and her inability to chew she has to be fed minced food. Massive caries and enamel defects from trismus required general dental repair and reconstruction under anesthesia. She visits a secondary school for disabled children, and communicates through a voice computer by saying “yes” or “no” or by making hand gestures.

Cranial MRI at the age of 1.5 years showed Leigh-like lesions with increased T2-signal intensity in the *putamen* and *globus pallidus* which became more prominent until age 2.2 years (Fig.3C). Proton spectroscopy in the basal ganglia showed an increased lactate peak. Laboratory examinations revealed mildly increased urine excretion of lactate and lactic acidemia (2.5–4.0 mmol/L). The amino acid profile was normal in cerebrospinal fluid (CSF) and plasma. CPK was normal and muscle biopsy at 2 years of age only showed unspecific myopathic changes. Biochemical investigation revealed normal activities for the OXPHOS complexes, CS, PDHc as well as in vitro pyruvate oxidation.

Patient #52236 (F10, II:1, c.[229G>C];[476A>G], p.[Glu77Gln];[Gln159Arg]), a girl, is the first child of healthy unrelated German parents. After a normal pregnancy, she was born at 42 weeks of gestation with normal birth measurements (weight 3120 g, length 51 cm). Motor development was considered normal during the first months of life, and she started walking at the age of 11 months. From this age, however, short episodes of fist clenching, teeth gnashing, horizontal nystagmus and unresponsiveness were noted. Although repeated EEG did not show epileptic discharges, treatment with phenytoin was started and maintained until age 5 years when the attacks disappeared. From age 17 months, progressive gait abnormalities were observed. These were episodic in the beginning, but evolved into a paraspastic gait in early childhood, and the girl became wheelchair-dependent at age 9.5 years. From age 2 years, visual problems became evident, and examination showed bilateral optic atrophy. Visual acuity at age 13 years was 1/50 for both eyes. From age 7 years, progressive sensorineural deafness was found, and the patient was supplied with hearing aids at age 9.5 years. At the same age, mild mental retardation and dysarthria were noted. The patient completed a secondary school for blind children, and has been working in a workplace for disabled persons from age 18 years.

Examination at age 31 years showed short stature (155 cm) and low body weight (40 kg). Visual acuity was off-chart, the patient could only notice hand movements, and there was a pendular nystagmus of the blind. Communication was impeded by severe dysarthria. She had generalized dystonia and a spastic tetraparesis, leading to an inability to walk independently. Sensation was entirely normal.

Electromyography (EMG), neurography and repeated EEG were normal. Laboratory examinations showed mildly elevated lactate (2.5 mmol/L, normal <1.8 mmol/L) in CSF and inconsistently in serum (2.0–2.8 mmol/L, normal <2.2 mmol/L). A muscle biopsy at age 9 years showed some type II fiber atrophy due to immobilization and a faint accumulation of lipid droplets in single fibers. There were no ragged red fibers (RRF) and no cytochrome *c* oxidase (COX) deficiency, and biochemical analysis of respiratory chain activities showed normal results. Brain MRI at age 15 years showed exclusively Leigh-like signal hyperintensities in the *caput nuclei caudati* and *putamen* bilaterally (Fig.3D). MR spectroscopy of the left *putamen* showed no elevation of lactate.

### Genetic analysis

We used a dual approach of exome sequencing and panel sequencing to analyze a cohort of patients with suspected mitochondrial disorders. Seven index patients (#52236, #57277, #68552, #68761, #73663, #76656, and #MRB166) were investigated by exome sequencing in Germany as described previously.^8^,^9^ In brief, we used a SureSelect Human All Exon 50 Mb Kit (Agilent) for enrichment and a HiSeq2500 (Illumina) for sequencing. Reads were aligned to the human reference assembly (hg19) with BWA (version 0.5.8 Open source software, Wellcome Trust Sanger Institute). More than 90% of the exome was covered at least 20× allowing for high-confidence variant calls. Single-nucleotide variants (SNVs) and small insertions and deletions were detected with SAMtools (version 0.1.7 Open source software, Wellcome Trust Sanger Institute). Variant prioritization was performed based on the autosomal-recessive patterns of inheritance and the notion that the clinical mitochondrial phenotype of the patients is very rare. We therefore excluded variants with a frequency higher than 0.1% in 3850 control exomes and public databases and focused on genes encoding mitochondrial proteins^10^ carrying two potentially pathogenic DNA variants. In two other patients (#346 and #376) we used a TruSeq Exome kit from Illumina. A detailed description of the bioinformatic pipeline and variant filtering used for these two cases has been published recently.^11^

In patient #42031 we applied targeted enrichment of 1476 nuclear genes (including 1013 genes coding for mitochondrial proteins according to the MitoCarta) using an in-solution hybridization capture method (NimbleGen Madison, WI, USA). Paired-end sequencing was performed on a HiSeq2500 (Illumina) to an average 178× coverage. Sequence alignment and variant calling was done with CLC Workbench v.7.0.4. CLC bio, Aarhus, Denmark

We used Sanger sequencing to confirm all identified *ECHS1* mutations and to test the carrier status of available family members. Primer sequences and PCR conditions are available upon request.

### Western blotting

Immunoblotting was performed using two different protocols. For the analysis of F2;II:1 (#42031), F10, II:2 (#52236), F9,II:2 (#57277), F5, II:3 (#73663) 45 *μ*g of protein of patients’ fibroblast cell homogenates were separated on 13% sodiumdodecyl sulfate polyacrylamide gel electrophoresis (SDS-PAGE) along with a protein standard (Precision Plus Protein Kaleidoscope, Biorad Hercules, CA, USA) and blotted onto polyvinylidine fluoride membranes (Immobilon, Merck Millipore Darmstadt, Germany). Primary antibodies were as follows: ECHS1 (66117, Proteintech Chicago, IL, USA), *β*-actin (ab13822, Abcam Cambridge, UK). Chemiluminescence detection was performed on an Odyssey Biomedical: Phoenix, AZ, USA infrared imaging system according to the manufacturer's instructions. For the analysis of F1, II:2 (#346), and F6, II:1 (#376) mitochondria were isolated by homogenization of cells in mitochondrial isolation buffer (20 mmol/L HEPES, 4-(2-hydroxyethyl)-1-piperazineethanesulfonic acid pH 7.4, 220 mmol/L mannitol, 70 mmol/L sucrose, 1 mmol/L Ethylenediaminetetraacetic acid) with 2 mg/mL Bovine serum albumin. Cell homogenates were centrifuged at 700*g* at 4°C for 5 min and postnuclear supernatant was collected. Mitochondria were pelleted by centrifugation at 10,000*g* at 4°C for 5 min and mitochondrial pellet was resuspended in mitochondrial isolation buffer for subsequent size-separation on 10% gels by SDS-PAGE, and transfer to a methanol-activated PVDF membrane. Immunoblotting was performed with primary antibodies from GeneTex (ECHS1) and Abcam (HSP60). Chemiluminescence detection was achieved with ECL Select (GE Healthcare, Little Chalfont, UK) and the membrane was viewed with the LAS4000 (GE Healthcare, Little Chalfont, UK), according to the manufacturer's instructions.

### Analysis of 2-enoyl-CoA hydratase activity

2-enoyl-CoA hydratase (ECHS1) activity was measured spectrophotometrically in fibroblast cell lysates following the absorbance of the unsaturated substrate crotonyl-CoA over time (15 min) as described.^12^,^13^ Briefly, trypsinized fibroblasts were resuspended in reduced Triton-X-100 (0.2%) and lysed by sonification. Lysates were centrifuged at 700*g* for 5 min to remove cellular debris and protein concentrations were determined in supernatants by the BCA method. The reaction mixture consisted of: 100 mmol/L potassium phosphate buffer (pH 8) for a final concentration of, 0.1 mg/mL BSA and 50 *μ*L of cell homogenate for a total of 15 *μ*g of protein. The reaction was started with 50 *μ*L of 250 *μ*mol/L crotonyl-CoA for a final concentration of 25 *μ*mol/L. Samples were measured at 37°C in UV cuvettes at 263 nm in a Shimadzu spectrophotometer. Enzymatic activity was calculated using a molar extinction coefficient of 6700. Enzymatic activity was normalized to the mitochondrial marker enzyme CS as previously described.^14^ All reactions were performed in at least three independent experiments in triplicates with similar findings.

### Palmitate loading assay

Palmitate loading in human skin fibroblasts of ECHS1 patients, healthy volunteers as negative controls and fibroblasts of patients with short- and medium-chain acyl-CoA dehydrogenase (MCAD) deficiency as disease controls were performed as previously described.^15^ In brief, cell cultures were maintained in Dulbecco's modified Eagle medium (Invitrogen, Darmstadt, Germany) supplemented with 2 mmol/L glutamine, 10% fetal calf serum, 1% penicillin/streptomycin, and Fungizone™ (all from Invitrogen) at 37°C and 5% CO_2_ in a humidified atmosphere until confluency. Palmitate loading was performed in serum- and glutamine-free minimal essential medium (Invitrogen) containing palmitic acid (200 *μ*mol/L; Sigma-Aldrich Taufkirchen, Germany), l-carnitine (400 *μ*mol/L; Sigma), and bovine serum albumin (0.4%; fatty acid-free; Sigma). After the end of the 96 h-incubation period, 10 *μ*L aliquots of the cell culture media were collected and mixed with isotope-labeled internal standard (Cambridge Isotope Laboratories, Tewksbury, MA 01876) in methanol. Twenty-five microliters of the butylated sample were used to analyze the acylcarnitine profile by tandem mass spectrometry. For each sample at least two independent measurements were performed. The acylcarnitine concentration was normalized to the protein content of the fibroblast sample and expressed as nmol/mg protein.

For the analysis of palmitate-dependent mitochondrial respiration, control and patient fibroblasts were seeded at a density of 20,000 cells/well in 80 *μ*L of high glucose Dulbecco's modified media (Gibco:Life Technologies, Darmstadt, Germany) supplemented with 10% fetal bovine serum (Invitrogen), 1% penicillin-streptomycin (Invitrogen) and 200 *μ*mol/L uridine (Sigma) in a XF 96-well cell culture microplate (Seahorse Bioscience, North Billerica, MA, USA) and incubated overnight at 37°C in 5% CO_2_. Culture medium was replaced with 160 *μ*L of serum- and glutamine-free minimal essential medium (Invitrogen) supplemented with l-carnitine (400 *μ*mol/L; Sigma), and bovine serum albumin (0.4%; fatty acid-free; Sigma) and cells were incubated for 24 h at 37°C in 5% CO_2_. Palmitate-BSA complex was prepared according to the manufacturer's protocol (Seahorse Bioscience). Oxygen consumption rate (OCR) was measured using a XF16 Extracellular Flux Analyzer 2 (Seahorse Biosciences). OCR was determined with no additions; after addition of BSA (30 *μ*mol/L) or palmitate-BSA conjugate (180/30 *μ*mol/L).

## Results

### Exome sequencing identifies *ECHS1* mutations

We applied whole exome sequencing in a cohort of 435 individuals with a suspected disorder of mitochondrial energy metabolism and identified six unrelated affected individuals carrying two heterozygous or homozygous rare variants (minor allele frequency [MAF] <0.1%) in *ECHS1* (Fig.2). Applying the same variant selection criteria to our in-house exome datasets from 3850 patients referred with presumed nonmitochondrial phenotypes revealed rare recessive-type *ECHS1* variants in one additional index case, F8, II.1 (#MRB166, Fig.2). This patient was investigated in the context of an intellectual disability study but the relevance of the *ECHS1* variants was hitherto unclear. Clinical follow-up and review of medical reports revealed, however, that the neurologic features of this patient (deafness, seizures, hypotonia, ataxia, and developmental delay) are very well compatible with a mitochondrial disorder. In contrast, it remains unclear whether the dysmorphic signs in this patient are related to the ECHS1 deficiency as dysmorphism is rather uncommon in mitochondrial diseases and was not found in any of the other ECHS1-deficient patients. The enrichment of rare biallelic *ECHS1* variants in genomes of individuals with a suspected mitochondrial disorder (*n* = 7) compared to 3850 control genomes was genome-wide significant (*P* < 1.1 × 10^−7^, Fisher exact test). In addition, in several individuals, *ECHS1* was the only gene coding for proteins with a predicted or confirmed mitochondrial localization harboring two rare DNA variants. We gained further evidence for a disease association of *ECHS1* by exome sequencing of 180 Japanese individuals with suspected mitochondrial disorders which identified two additional *ECHS1-*mutant individuals, F1, II.2 (#346) and F6, II.1 (#376, Fig.2). By panel sequencing in a further patient with a similar clinical presentation we identified two heterozygous *ECHS1* mutations in F2, II:1 (#42031, Fig.2). The combined approaches of exome and candidate gene sequencing identified a total of 13 different disease alleles in ten index patients. Pedigrees of investigated families and localization of identified *ECHS1* mutations as well as the evolutionary conservation of affected amino acid residues are shown in Figure2B. All *ECHS1* mutations were confirmed by Sanger sequencing in the index patients. Carrier testing of available parental samples confirmed a biallelic localization of the mutations. DNA from the parents was not available in families F4 and F6, however, in the latter a compound heterozygous state of the mutations was confirmed by haplotype phasing. The healthy siblings of families F10, F9, F3, and F7 were either wild-types or heterozygous carriers. For families F3, F5, and F1, family history was positive for the occurrence of similar clinical conditions but no DNA samples were available for molecular tests from individuals F1, II:1 and F3, II:3.

Of the 13 different *ECHS1* mutations identified in our cohort, only one, c.431dup, p.Leu145Alafs*6, predicts a premature truncation and loss of function of the protein (Table S1). All other detected mutations are missense variants, indicating that a complete loss of ECHS1 function may be embryonic lethal. With the exception of the c.268G>A, p.Gly90Arg variant, all are predicted to be disease-causing (MutationTaster2).^16^ Although all variants are rare (MAF < 0.05%), three mutations were identified in more than one family (c.161G>A, p.Arg54His, *n* = 3; c.176A>G, p.Asn59Ser, *n* = 2; and c.476A>G, p.Gln159Arg, *n* = 2), in each case with different mutations on the other allele. In addition, the c.476A>G, p.Gln159Arg mutation was found in the homozygous state in a third, consanguineous family. However, there is no mutational hotspot and the identified mutations are distributed across all eight coding exons of *ECHS1* except for exons 1 and 7 (Fig.2B).

### Functional consequences of *ECHS1* mutations

Next, we analyzed the consequences of *ECHS1* mutations in fibroblast cell lines. In six cell lines, we investigated ECHS1 protein levels and in four of them we measured 2-enoyl-CoA hydratase activity. SDS-PAGE separation of mitochondrial fractions and total cell lysates followed by immunodetection with an antibody against ECHS1 revealed a severe decrease in ECHS1 steady-state levels in all patient-derived fibroblast cell lines. To test the functional impact of *ECHS1* mutations, we determined 2-enoyl-CoA hydratase activity in the fibroblasts cell lysates from individuals F2, II.1 (#42031), F10, II:2 (#52236), F9, II:2 (#57277), and F5, II:3 (#73663). The 2-enoyl-CoA hydratase activity was markedly reduced in patients’ cell lines with residual activities varying between 14% and 50% of controls (Fig.3B).

Palmitate loading in fibroblasts of three ECHS1 patients induced an acylcarnitine profile very similar to that of short-chain acyl-CoA dehydrogenase (SCAD) deficiency. The characteristic metabolite, butyrylcarnitine, increased to 170%, 199%, or 273% of the upper normal range of the controls. In another ECHS1-deficient patient, however, butyrylcarnitine was within the range of controls (48% of the upper normal range). In comparison, butyrylcarnitine increased to 388% of controls in patient fibroblasts with SCAD deficiency and 44% in MCAD deficiency. These results indicate that ECHS1 deficiency results in a mild functional disorder of short-chain fatty acid oxidation.

**Figure 4:**
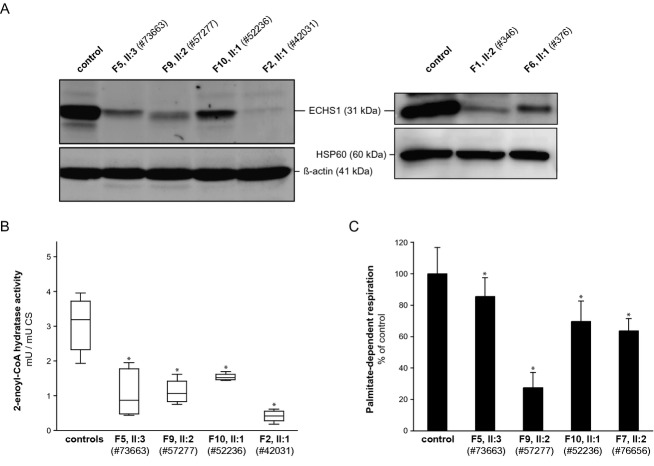
Analysis of ECHS1 levels and enzymatic activity. (A) Analysis of ECHS1 steady state levels by immunoblotting show a decrease in the amount of ECHS1 in patient-derived cell lines compared to controls. (B) Analysis of residual ECHS1 enzymatic activity indicates reduced 2-enoyl-CoA hydratase activity in cell lysates from patients’ fibroblasts compared to controls. Results shown are from at least three experiments performed in triplicates and controls (*n* = 4). Boxplot whiskers indicate range from 5th to 95th percentile. *P < 0.001; two-tailed unpaired *t*-test. (C) Analysis of palmitate-dependent OCR in fibroblast cell lines revealed impaired respiration in patients’ cells in comparison to controls. The experiment was performed several times with very similar results. The data are shown from one experiment performed with more than 10 replicates for each cell line grown and treated in parallel. Error bars indicate 1 SD. *P < 0.001; two-tailed unpaired *t*-test. ECHS1, short-chain enoyl-CoA hydratase; OCR, oxygen consumption rate.

This notion is further supported by the observation of reduced palmitate-dependent respiration in patient-derived fibroblasts compared to controls (Fig.4C). Noteworthy, in the three cell lines tested for both 2-enoyl-CoA hydratase activity and palmitate-dependent respiration, the severity of defects correlated. Together, these data provide evidence for the pathogenicity of eleven *ECHS1* alleles.

### Organic acids in urine

In analogy, organic acid analysis by gas chromatography/mass spectrometry in urine of patient #73663 revealed slightly elevated concentrations of ethylmalonate, a key metabolite of SCAD deficiency. Functional deficiency of short-chain fatty acid oxidation was also shown by intermittently elevated plasma fatty acids (250–780 *μ*mol/L; normal <300 *μ*mol/L) with concomitantly low normal plasma ketone bodies (65–90 *μ*mol/L; normal <200 *μ*mol/L) in postprandial state of some patients. However, ketogenesis was only mildly impaired which was shown by a mild increase in ketone bodies during catabolism (570–630 *μ*mol/L, normal <3000 *μ*mol/L). In addition, we identified 2-methyl-2,3-dihydroxybutyrate which likely derives from acryloyl-CoA or, alternatively, methacrylyl-CoA, both metabolites of valine oxidation, in urines of four ECHS1 patients. This metabolite correlated with disease severity as its concentration was 229-fold higher than the median value in 35 controls in the severely affected patient #42031 (onset at birth, died at 11 months), 39-fold higher in patient #73663 (onset on day 5, alive at 2 years), sixfold higher in patient #76656 (onset at 2 years, alive at 5 years), and even in the normal range in the mildest affected case #52236 (onset at 11 months, alive at 31 years), the only patient who survived into adulthood.

### Phenotypic features of ECHS1 deficiency

In contrast to gene identification strategies in which an a priori phenotypic stratification of the investigated cohort was the main key to success,^17^ this group of *ECHS1*-mutant individuals was identified by exome sequencing of a large cohort of individuals with suspected mitochondrial disorders. This approach allows for a rather unbiased assessment of the phenotypic spectrum associated with ECHS1 deficiency.

Disease severity ranged from presentations with neonatal onset and death in early infancy to survival into adulthood. In nine of 10 affected individuals, pregnancy was normal and the children were born at term with normal body measurements. First clinical signs appeared in the prenatal period in one individual (oligohydramnios) and in the neonatal period (first month) in seven. Presenting signs included epileptic seizures, muscular hypotonia, respiratory and cardiac failure, failure to thrive, elevated lactate, and metabolic acidosis. In the two mildest affected individuals, developmental delay (F9, II:2, #57277) or episodic neurological symptoms (F10, II:1, #52236) were noted in the first year of life and lactate elevations were only intermittent and mild. While six of 10 affected individuals are still alive at the age of 2, 3, 5, 8, 16, and 31 years, four died at the age of 4, 11, 28, and 90 months, respectively. Interestingly, cardiomyopathy was only found in the individuals with a severe course and early death and none of the patients over 3 years of age.

In the course of the disease, neurological signs are most prominent including sensorineural deafness (9/9), developmental delay (8/10), epileptic seizures (6/9), dystonia (5/9), optic atrophy (6/10), muscular hypotonia (6/10), and spastic paresis (2/10). Other clinical features include cardiomyopathy (4/10) and respiratory failure (3/10). Increased blood, urine, and CSF lactate levels were found in seven of 10 individuals. Brain MRI was performed in nine of 10 individuals and showed white matter changes and brain atrophy in the very severe, early onset cases (*n* = 5) and Leigh-like bilateral T_2_ hyperintensities in the basal ganglia (nucleus caudatus, putamen, and globus pallidus) in the less severely affected individuals (*n* = 4; Fig.3). MR spectroscopy of basal ganglia showed elevated lactate concentrations in three of seven individuals.

Taken together, our findings identified *ECHS1* mutations as a cause of a new clinical entity characterized by an early onset, very severe (Leigh-like) mitochondrial encephalopathy with deafness, epilepsy, optic atrophy, and cardiomyopathy. Consistently elevated plasma concentrations of lactate indicate a dysfunction of the mitochondrial energy metabolism.

In view of the triad of (1) a severe progressive encephalopathy, (2) associated with bilateral basal ganglia lesions in MRI and autopsy, and (3) the mitochondrial dysfunction, ECHS1 deficiency is a new Leigh-like syndrome but can be differentiated from other forms of Leigh syndrome clinically and biochemically.

### Analysis of OXPHOS enzymes in muscle tissue and fibroblasts

Since the clinical presentation suggested a mitochondrial disorder, eight of 10 patients received muscle and/or skin biopsies with detailed analysis of respiratory chain complexes, pyruvate dehydrogenase complex, ATP production, and pyruvate oxidation. Despite the severe clinical presentation of ECHS1 patients, only four of them showed mild and inconsistent changes in pyruvate oxidation (patient #42031), ATP production (patient #68761), and decreased activities of NADH:CoQ oxidoreductase (complex I of the respiratory chain) or cytochrome *c* oxidase (complex IV of the respiratory chain). Noteworthy, all these patients died (age of death: 4 months, 11 months, 3 years, 7.5 years), whereas four patients without documented changes in these analyses (#52236, #57277, #73663, #76656) have survived until now (age at latest visit: 2, 5, 16, and 31 years of life).

## Discussion

ECHS1 is a mitochondrial matrix enzyme that is involved in several metabolic pathways including fatty acids and amino acids. Deficiency of this enzyme is expected to result in impaired mitochondrial fatty acid oxidation. However, as the membrane-bound enzymatic machinery of long-chain fatty acids remains intact, ketogenesis should not be completely disrupted in ECHS1-deficient individuals and, assumingly, the resulting clinical phenotype should be relatively benign – similar to SCAD deficiency.^18^,^19^ In fact, ECHS1-deficient individuals showed mild to moderate hypoketosis, but did neither present with hypoketotic hypoglycemia and hypoglycemic encephalopathy nor were acylcarnitine profiles in blood suggestive of a mitochondrial *β*-oxidation disorder. Deficiency of short-chain fatty acid oxidation, however, was unmasked by palmitate loading of ECHS1-deficient fibroblasts (Fig.4C). This metabolic challenge induced a selective increase in butyrylcarnitine in three of four patients tested, which in combination with slightly increased ethylmalonate in urine reflects impaired oxidation of short-chain fatty acids.

Despite this relatively mild metabolic derangement (concerning mitochondrial *β*-oxidation), ECHS1-deficient individuals presented with a severe clinical phenotype, with a high frequency of Leigh-like syndrome, neonatal lactic acidosis, sensorineural hearing loss, muscular hypotonia, cardiomyopathy, and respiratory failure. Although this combination of clinical findings, in particular the leading neurological presentation, is rather uncommon for fatty oxidation defects,^20^,^21^ there is some clinical overlap with long-chain acyl-CoA dehydrogenase (LCHAD) deficiency and mitochondrial trifunctional protein (MTP) deficiency which resemble a primary OXPHOS deficiency. Affected individuals with LCHAD and MTP deficiency show a high frequency of neurological signs and symptoms including developmental delay, muscular hypotonia, epilepsy, and lipid storage myopathy as well as cardiomyopathy and sudden death in newborns and infants.^22^,^23^ Similar to ECHS1 deficiency, elevated lactate is also often found in LCHAD/MTP deficiency suggesting mitochondrial dysfunction.^22^,^23^ In contrast to ECHS1 deficiency, however, individuals with LCHAD/MTP deficiency often present with hepatic dysfunction, retinopathy and peripheral neuropathy, but usually lack dystonia and sensorineural hearing loss. Interestingly, accumulating long-chain fatty acids and acyl-CoA are thought to act as mitochondrial toxins inhibiting energy metabolism in individuals with LCHAD and MTP deficiency.^24^,^25^

The striking discrepancy between an expected moderate impairment of mitochondrial fatty acid oxidation and the severe clinical presentation of ECHS1-deficient individuals argues for an additional pathomechanism. In addition to its function in fatty acid oxidation, ECHS1 has also been suggested to be involved in the l-isoleucine, l-valine and l-lysine oxidation using tiglyl-CoA, 2-methacrylyl-CoA or crotonyl-CoA, respectively, as a substrate (Fig.1).^26^ 2-Methacrylyl-CoA is a highly reactive compound and readily undergoes reactions with free sulfhydryl groups thereby inactivating important sulfhydryl-containing enzymes such as respiratory chain complexes and pyruvate dehydrogenase complex.^26^ Accumulation of 2-methacrylyl-CoA has been considered as toxic compound in HIBCH deficiency, a disorder of the fifth step of valine oxidation with Leigh-like neurological phenotype and combined deficiency of multiple mitochondrial enzymes (Fig.1).^4^,^5^ The neurological phenotypes of individuals with ECHS1 deficiency and HIBCH deficiency are strikingly similar. As ECHS1 is suggested to catalyze the fourth step of valine oxidation, accumulation of 2-methacrylyl-CoA is expected and may be responsible for some of the pathological changes. Indeed, very recently, *ECHS1* mutations were reported in two siblings with Leigh disease and remarkable clinical and biochemical similarities to HIBCH deficiency,^6^ with most prominent elevations of methacrylate and acrylate metabolites indicating deficiency of valine oxidation. Our observation of elevated levels of 2-methyl-2,3-dihydroxybutyrate in the urinary organic acids, a likely derivative of valine oxidation metabolites, provides further evidence for the involvement of defective valine metabolism in the pathogenesis of the disease. It may, in addition, be a useful biomarker of disease course as we observed some correlation already with the disease severity. Despite the clinical and biochemical similarities between ECHS1 and HIBCH deficiency, patients with ECHS1 deficiency showed less often impaired oxidative phosphorylation than those with HIBCH deficiency in the investigated fibroblasts or muscle biopsy samples. However, those ECHS1 patients with detectable OXPHOS deficiency had a severe course of disease and died during infancy or early childhood.

In conclusion, the identification of 10 unrelated individuals with *ECHS1* mutations allowed us to define the phenotypic spectrum of this new mitochondrial disease entity which can be differentiated clinically and biochemically from other molecular causes of Leigh or Leigh-like syndrome. Regarding pathogenesis, we found both a *β*-oxidation defect and impaired valine oxidation. We speculate that both defects contribute to the clinical and biochemical phenotype of this newly defined disorder. Intriguingly, ECHS1 deficiency may be amenable to treatment, provided that the elevation of toxic 2-enoyl-CoA compounds can be influenced by dietary intake. All possibly affected amino acids (valine, isoleucine, and lysine) in ECHS1 deficiency are essential amino acids and their dietary uptake can be expected to influence their catabolism. In addition, maintenance of high glucose levels, as recommended in fatty acid oxidation defects might be protective for the heart in ECHS1 deficiency. Anyway, further studies – preferably with an ECHS1 animal model – are necessary to unravel the exact pathomechanisms in ECHS1 deficiency and the efficacy and safety of possible treatments.

## Supporting Information

**Table S1.** Annotation details on identified ECHS1 mutations.
