# Supplementary material for: Deficiency of ECHS1 causes mitochondrial encephalopathy with cardiac involvement
Source: Ann Clin Transl Neurol. 2015 Mar 13;2(5):492–509. doi: 10.1002/acn3.189 (PMC4435704; doi:10.1002/acn3.189)
Supplement: Supplementary file 1 — Table S1. Annotation details on identified ECHS1 mutations. [file acn30002-0492-sd1.docx]

| **Supplementary Table 1. Annotation details on identified *ECHS1* mutations** | | | | | | | | | | |
| --- | --- | --- | --- | --- | --- | --- | --- | --- | --- | --- |
| *ECHS1* mutations | | |  | Frequency ExAC (>120,000 alleles) | |  | Annotation/Prediction | | | |
| Chromosomal Position  (hg19) | cDNA (NM_004092.3) | Protein (NP_004083.3) |  | Allele Count (Heterozygotes) | Allele Frequency |  | Annotation | Polyphen | SIFT | MutationTaster |
| NC_000010.10:g.135184252G>A | c.98T>C | p.Phe33Ser |  | - |  |  | missense | probably_damaging | damaging | disease causing |
| NC_000010.10:g.135184189C>T | c.161G>A | p.Arg54His |  | 1 | 8.256e-06 |  | missense | probably_damaging | damaging | disease causing |
| NC_000010.10:g.135184174T>C | c.176A>G | p.Asn59Ser |  | 5 | 4.133e-05 |  | missense | probably_damaging | damaging | disease causing |
| NC_000010.10:g.135184153A>G | c.197T>C | p.Ile66Thr |  | - |  |  | missense | benign | damaging | disease causing |
| NC_000010.10:g.135184121C>G | c.229G>C | p.Glu77Gln |  | - |  |  | missense | benign | damaging | disease causing |
| NC_000010.10:g.135184082C>T | c.268G>A | p.Gly90Arg |  | - |  |  | missense | benign | damaging | polymorphism |
| NC_000010.10:g.135183428C>T | c.394G>A | p.Ala132Thr |  | - |  |  | missense | probably_damaging | damaging | disease causing |
| NC_000010.10:g.135182510dup | c.431dup | p.Leu145Alafs*6 |  | - |  |  | frameshift | - | - | disease causing |
| NC_000010.10:g.135182492T>C | c.449A>G | p.Asp150Gly |  | - |  |  | missense | probably_damaging | damaging | disease causing |
| NC_000010.10:g.135182465T>C | c.476A>G | p.Gln159Arg |  | 14 | 0.0001148 |  | missense | benign | tolerated | disease causing |
| NC_000010.10:g.135180429C>T | c.583G>A | p.Gly195Ser |  | 1 | 8.229e-06 |  | missense | probably_damaging | damaging | disease causing |
| NC_000010.10:g.135179546A>G | c.673T>C | p.Cys225Arg |  | 1 | 8.156e-06 |  | missense | probably_damaging | damaging | disease causing |
| NC_000010.10:g.135176428T>C | c.817A>G | p.Lys273Glu |  | 3 | 2.445e-05 |  | missense | benign | damaging | disease causing |
